# Footprint-Aware Power Capping for Hybrid Memory Based Systems

**DOI:** 10.1007/978-3-030-50743-5_18

**Published:** 2020-05-22

**Authors:** Eishi Arima, Toshihiro Hanawa, Carsten Trinitis, Martin Schulz

**Affiliations:** 8 School of Computing, University of Utah, Salt Lake City, UT USA; 9 Cray, a Hewlett Packard Enterprise Company, Seattle, WA USA; 10 Helmholtz-Zentrum Dresden-Rossendorf, Dresden, Germany; 11 Extreme Computing Research Center, King Abdullah University of Science and Technology, Thuwal, Saudi Arabia; 12 The University of Tokyo, Tokyo, Japan; 13 Technical University of Munich, Munich, Germany

## Abstract

High Performance Computing (HPC) systems are facing severe limitations in both power and memory bandwidth/capacity. By now, these limitations have been addressed individually: to improve performance under a strict power constraint, power capping, which sets power limits to components/nodes/jobs, is an indispensable feature; and for memory bandwidth/capacity increase, the industry has begun to support hybrid main memory designs that comprise multiple different technologies including emerging memories (e.g., 3D stacked DRAM or Non-Volatile RAM) in one compute node. However, few works look at the combination of both trends.

This paper explicitly targets power managements on hybrid memory based HPC systems and is based on the following observation: in spite of the system software’s efforts to optimize data allocations on such a system, the effective memory bandwidth can decrease considerably when we scale the problem size of applications. As a result, the performance bottleneck component changes in accordance with the footprint (or data) size, which then also changes the optimal power cap settings in a node. Motivated by this observation, we propose a power management concept called  and a profile-driven software framework to realize it. Our experimental result on a real system using HPC benchmarks shows that our approach is successful in correctly setting power caps depending on the footprint size while keeping around 93/96% of performance/power-efficiency compared to the best settings.

## Introduction

Power consumption has become the major design constraint when building supercomputers or High Performance Computing (HPC) systems. For instance, the US DOE once had set a power constraint of 20 MW per future exascale system to ensure their economical feasibility. To achieve orders of magnitude performance improvement under such a strict power constraint, we must develop sophisticated power management schemes. To this end, *power capping* (setting a power constraint to each job/node/component) and *power shifting* (shifting power among components depending on their needs under a given power budget) are promising and the most common approaches [5, 9, 20, 27, 28, 31, 33].

At the same time, we continue to face limited memory bandwidths and capacities in HPC systems. On the one hand, to improve bandwidth, architecting main memories with 3D stacked DRAM technologies, such as HBM [36] and HMC [6], is an attractive approach. However, these technologies have limited capacity-scalability compared to conventional DDR-based DRAM [16]. On the other hand, using emerging scalable NVRAMs (Non-Volatile RAMs, e.g., PRAM [8, 19, 26, 30], ReRAM [2], STT-MRAM [3, 18, 23] and 3D Xpoint memory [14]) are promising in terms of capacity, but these technologies are generally much slower than conventional DRAM. As a consequence, the industry has been shifting toward hybrid memory designs: main memories with multiple different technologies (e.g., 3D stacked DRAM + DDR-based DRAM [16] or DRAM + NVRAM [14]), which are usually heterogeneous in bandwidth and capacity.

Driven by these trends, this paper focuses on a power management technique explicitly tailored for such hybrid memory based systems. Our approach is based on the following observation: when we scale the problem size (e.g., by using finer-grained and/or larger-scaled mesh models for scientific applications), the performance bottleneck can change among components. As a result, the optimal power budget settings also change due to this *bottleneck shifting* phenomenon. Thus, to exploit higher performance under a power constraint, we should also *shift power* between CPU and memory system in accordance with the *footprint (or data) size* of applications, which we call  in this paper. As we often use various problem settings for each scientific application, this footprint awareness is critically important.

To realize the concept of FPCAP, we first formulate the power allocation problem and provide a regression-based performance model to solve it. Then, based on the formulations, we present a profile-based software framework that optimizes the power allocation to each component based on an efficient offline model-fitting methodology as well as an online heuristic algorithm. Our experimental results measured on a real system shows that our approach achieves near optimal allocations under various power caps.

The followings are the major contributions of this study:

- We demonstrate the bottleneck shifting phenomenon by scaling the problem size on a hybrid memory based system and propose a power management concept called FPCAP.
- We quantify its potential benefit using various mini HPC applications chosen from the CORAL benchmark suite.
- We formulate the power allocation problem and present an empirical performance model to solve it.
- Based on this formulation, we provide a profile-based software framework consisting of an efficient calibration method as well as an algorithm based on a hill climbing based heuristic.
- We evaluate our approach on a hybrid memory based system. The experimental result shows that our framework is successful in setting power caps to components in accordance with the footprint size.

## Background and Related Work

**Fig. 1.**
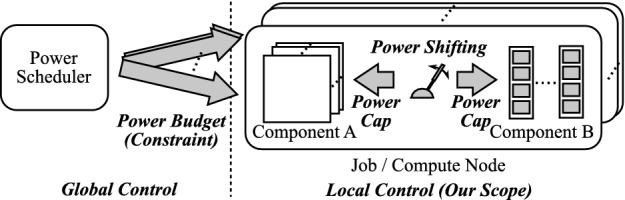
Assuming hierarchical power management

Various power management schemes for large-scaled systems have been proposed so far, and such schemes generally assume hierarchical power controls and can be classified into global or local parts. Figure 1 illustrates a typical power control hierarchy for them. In the figure, the power scheduler distributes power budgets or sets power constraints to nodes/jobs (*global control*). Then, in each node/job the allocated power is distributed to the components with the goal of maximizing performance by shifting power from non-bottleneck components to the bottleneck one (*local control*). Our paper belongs to the latter part and is the first work that (1) *focuses on the bottleneck shifting phenomenon when scaling the problem size on the hybrid memory based nodes* and (2) *provides a power allocation scheme based on the observation*.

The followings summarize the related work to ours.

**Global Power Controls: ** Since the power consumption of large-scaled systems have become a significant problem, various power scheduling schemes and implementations for them have been proposed so far [5, 9, 28, 31, 33]. These studies are usually based on the concept of overprovisioning: installing more hardware than the system can afford in terms of power, and intelligently controlling power supply to each job/node while keeping the total system power constraint [27]. Although these studies are very useful to improve the total throughput under the system power constraint, they focus on how to distribute power budgets across nodes/jobs and thus are orthogonal to ours.

**Local Power Controls: ** The concept of power shifting firstly appeared in [10], and power capping was proposed to enable power shifting [20]. Since then, various other local power management techniques have been proposed. However, ours is the first work in providing a way to optimize the power allocations to *CPU* and *hybrid memory system* in accordance with the *footprint size*. Several studies focused on power shifting between processors (CPU or GPU) and memories [7, 10, 12, 24, 29, 32], but they did not target hybrid memory systems. Others propose various approaches based on different concepts: power shifting in a NUMA node [11], CPU-GPU power optimizations [4, 17], power shifting between CPUs and networks [21, 22], and I/O-aware power shifting [35], which do not consider memories.

**Power Management for Hybrid Memory Systems: ** As DRAM scaling is at risk, many studies have focused on hybrid memory architectures, and some of them proposed power control schemes for them. H. Park et al. [26] uses DRAM as a cache in a DRAM-PRAM hybrid memory system and applies cache-decay, a power reduction technique that turns-off unused cachelines, to save the refresh power of DRAM. Other studies aim at optimizing data allocations on DRAM-PRAM hybrid memories to reduce the impact of the write access energy of PRAM [30, 39]. Although these approaches are promising, they still focus only on hybrid main memory systems—ours covers both memories and processors and optimizes power allocations to them. Moreover, these studies are based on architectural simulations, and thus most of them require hardware modifications, while ours works on real systems.

**Fig. 2.**
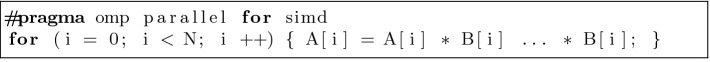
Tested synthetic streaming code (footprint size $\propto$ N, arithmetic intensity or simply AI $\propto$ the number of *B[i])

**Fig. 3.**
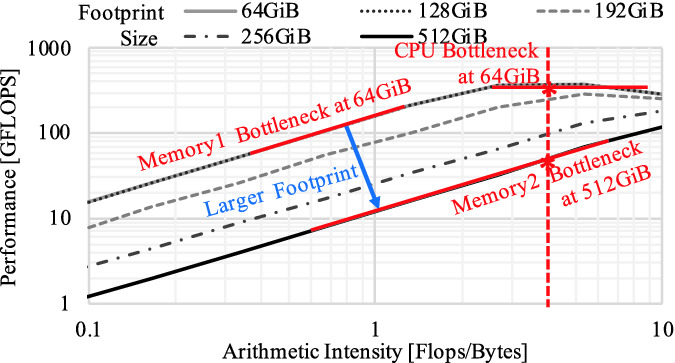
Measured rooflines [38]

**Fig. 4.**
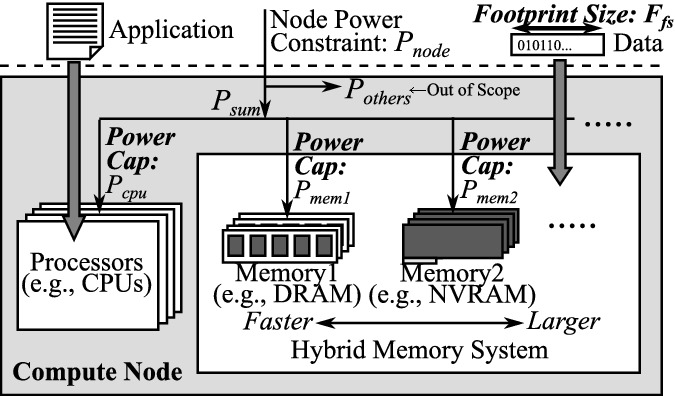
Concept of our proposal

## Motivation and Approach

The goal of this research is to provide a power management scheme suitable for emerging HPC nodes composed of *hybrid main memories* under a given node power constraint. When we execute scientific applications on HPC systems, we usually utilize various problem inputs, which can considerably change the *footprint size* (the memory consumption of the running application). For instance, we change the granularity/scale of mesh models and/or the number of time steps for scientific applications. Under such scenarios, *footprint-awareness* is essential to optimize the power settings of the components, which will be described in the following subsections.

### Motivation: Roofline Observation

We execute the synthetic streaming code shown in Fig. 2 on our hybrid memory based system whose configurations are provided in Sect. 6. In this experiment, we change the footprint size and the arithmetic intensity (or simply *AI*) of this application by scaling the array size (*N*) and the number of arithmetic operations ($* B[i]$). Figure 3 describes the results. The horizontal axis indicates the arithmetic intensity (Flops/Bytes), while the vertical axis shows the performance (GFLOPS). The shapes of the curves can be well-explained by the roofline model [38]: (1) for smaller arithmetic intensity, the performance is capped by the memory system bandwidth (the slope lines), which means *the memory system is the performance bottleneck*; (2) but for higher arithmetic intensity, it is limited by the CPU throughput (the horizontal lines)—in other words *the CPU is the performance bottleneck*.

In this evaluation, we observe the phenomenon of *bottleneck shifting*: although the system software attempts to optimize the data mapping on the hybrid main memory, the effective bandwidth decreases as the footprint size scales due to more frequent accesses to the large (but slow) memory, and as a result, the slope line in Fig. 3 moves toward the downside^1^. Because of this effect, *the performance bottleneck can shift from the CPU to the memory system* even for CPU intensive workloads when we increase the footprint size. As the fundamental principle of the power management for power constrained systems is *allocating more power budget on the bottleneck component*, thus focusing on this phenomenon is a pivotal approach.

### Concept: Footprint-Aware Power Capping

Driven by the above observation, we propose a power management concept called  that optimizes power allocations to CPUs/memories in a node depending on the ***footprint size (***$F_{fs}$ ***)*** as well as the application features under a given node power constraint ($P_{node}$) that is assigned by the power scheduler of the system. The concept is illustrated in Fig. 4. In this figure, we optimize the power budget allocations (or power caps) to the CPUs ($P_{cpu}$) and the Memory *i* ($P_{memi}$)$_{i=1,2,...}$ in accordance with these inputs. In the figure, $P_{others}$ shows the total power limits of the other components that are out of the scope of this paper, which we follow the prior node-level power management studies [7, 12, 32]. More specifically, we assume $P_{others}$ is reserved accordingly, and we focus on distributing the rest of the allocated node power budget $P_{sum} (= P_{node} - P_{others})$ to the CPUs and the memories under the constraint of $P_{cpu} + P_{mem1} + \cdots \le P_{sum}$.

### Performance Impact

Next, we demonstrate the potential performance benefit of FPCAP using our hybrid memory based system. More specifically, we observe how the optimal combination of {$P_{cpu}$, $P_{mem1}$, $P_{mem2}$} changes depending on the footprint sizes using Small or Large problems while keeping the total power cap at a constant value (here, we set $\sum P_x = P_{sum} = 260[W]$). At the same time, we also confirm the performance impacts of naive power allocations that do not consider the footprint size of applications. The details of the system settings as well as the workload specifications including the definitions of Small/Large problems will be provided later in Sect. 6.

**Fig. 5.**
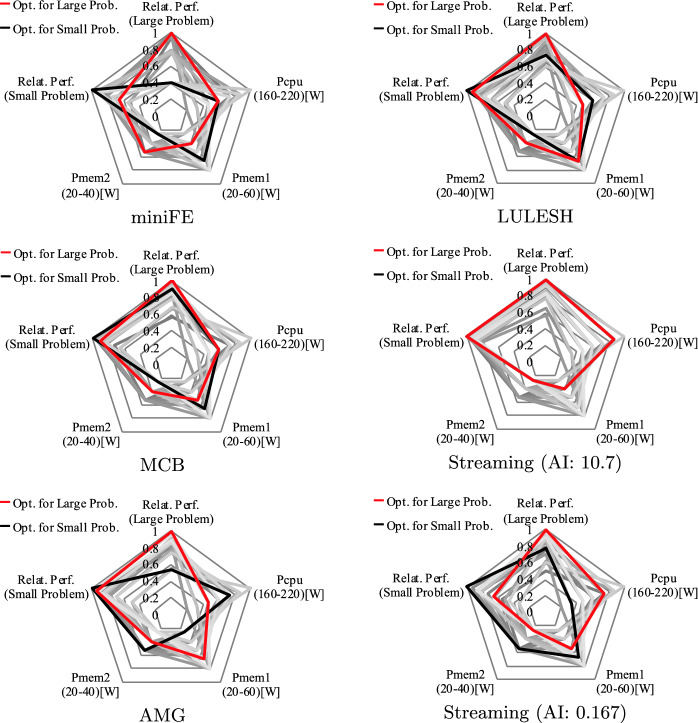
Performance comparison of various power allocation settings (constraint: $P_{sum}$ = 260[W]) for two different problem settings (Small/Large problems) (Color figure online)

**Fig. 6.**
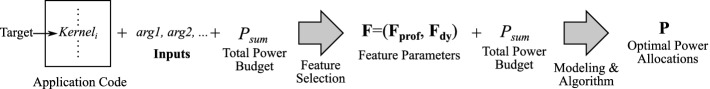
Overall parameters transformation

**Table 1.** Definitions of parameters/functions

Application related parameters	
*Kernel*	Target kernel in an application
$\mathbf {Inputs}$	Inputs for the application: $\mathbf {Inputs}=(arg1, arg2, \cdots )$
$\mathbf {F}$	Feature parameters that represent the kernel + inputs ($\mathbf {F}=(\mathbf {F_{prof}},\mathbf {F_{dy}})$)
$\mathbf {F_{prof}}$	Parameters obtained after a profile run (e.g., FP operations per instruction)
$\mathbf {F_{dy}}$	Parameters dynamically collected at runtime (e.g., **footprint size** $F_{fs}$)
Power related parameters	
$\mathbf {P}$	Vector of power allocations to components: $\mathbf {P}=(P_{cpu},P_{mem1},P_{mem2},\cdots )$
$P_{x}$	Allocated power budget to a component *x* $(x = cpu, mem1, mem2, \cdots )$
$S_{P_{x}}$	Set of power cap values for a component *x*: $P_x \in S_{P_x} (x = cpu, mem1, mem2, \cdots )$
$P_{sum}$	Given total power constraint
Objective functions	
$\mathrm {Obj}(\mathbf {P}, \mathbf {F})$	Objective function to be maximized (e.g., $\mathrm {Obj}(\mathbf {P}, \mathbf {F}) = \text {Perf}(\mathbf {P}, \mathbf {F})$)
$\mathrm {Perf}(\mathbf {P}, \mathbf {F})$	Performance as a function of $\mathbf{P}$ and $\mathbf{F}$
$\mathrm {PowEff}(\mathbf {P}, \mathbf {F})$	Power efficiency: $\mathrm {Perf}(\mathbf {P}, \mathbf {F})/\sum P_x$

Figure 5 illustrates the evaluation results for different applications. Each spider graph indicates the relative performance of two different problems along with the power cap settings for all the possible power combinations under the given total power constraint. Here, the performance is normalized to that of the optimal combination for each problem/application. In the figures, the optimal settings for Small/Large problems are highlighted with black/red lines.

Overall, the impact of power cap settings on performance is quite significant, and some cases also a slowdown can happen when the power allocations are not set accordingly. In addition, the optimal power allocations changes when we scale the problem sizes for most of the applications, thus FPCAP is effective.

For miniFE, LULESH and MCB allocating more power budgets on Memory 2 is effective when we scale the footprint sizes, which matches our roofline analysis provided in the last subsection. Also, the footprint size does not affect the performance bottleneck for very CPU intensive codes such as our synthetic code (Streaming (AI: 10.7)) described in Sect. 3.1, thus the optimal settings do not change for it when we change the problem size. For AMG and Streaming (AI: 0.167), reducing $P_{mem2}$ is effective when the footprint size is scaled. One major reason of this phenomenon is that the software-based data management adopted on our system—CPU also consumes power to handle the data transfers between Memory 1 and Memory 2, which can also change the performance bottleneck among the components.

## Formulation and Modeling

Motivated by the observation in the last section, we optimize the power allocations to components while taking the footprint size and other aspects into considerations (FPCAP). In this section, we firstly formulate the problem definition. Then, we provide a simple model to solve it.

### Problem Formulation

Figure 6 summarizes how parameters are transformed through our optimization. Our approach receives a kernel code region (*Kernel*), inputs for the applications such as arguments ($\mathbf {Inputs}$) that determine the footprint size ($F_{fs}$), and the total power constraint or budget ($P_{sum}$) set to the power capping targets within a node ($cpu, mem1, \cdots$). We then convert two of them (*Kernel* & $\mathbf {Inputs}$) into feature parameters ($\mathbf {F}$) that represent the behavior of the kernel executed with the inputs. The feature parameter vector is divided into profile-based statistic ($\mathbf {F_{prof}}$) and dynamically collected information ($\mathbf {F_{dy}}$), of which the latter includes the footprint size ($F_{fs}$). Finally, based on our modeling/algorithm provided later, we optimize the power caps to different components ($\mathbf {P}$).

This can be formulated as the following optimization problem:Here, we consider maximizing the objective function $Obj(\mathbf {P}, \mathbf {F})$ under the power constraint $P_{sum}$. This objective function can be performance ($Pref(\mathbf {P}, \mathbf {F})$), power efficiency ($PowEff(\mathbf {P}, \mathbf {F})$), or others. The power cap allocated to a component *x* is taken from a set of pre-determined power cap values $S_{P_x}$. Note that the functions and parameters used here are summarized in Table 1.

**Fig. 7.**
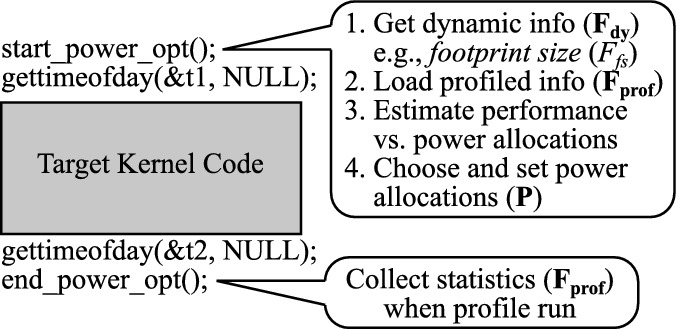
Kernel-level optimization

**Fig. 8.**
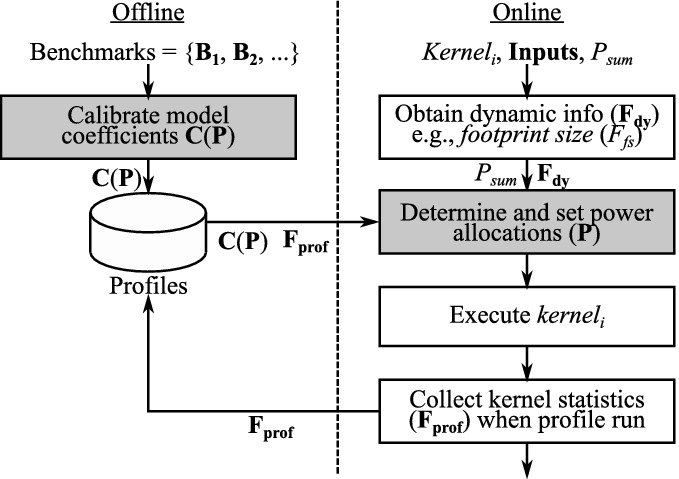
Framework overview

### Performance Model

In this study, we utilize a widely-used linear regression model for our performance estimation. More specifically, we estimate performance as follows:

1 $$\begin{aligned} Perf(\mathbf {P}, \mathbf {F}) = C_1(\mathbf {P})H_{1}(\mathbf {F}) + C_2(\mathbf {P})H_{2}(\mathbf {F}) + \cdots = \mathbf {C}(\mathbf {P})\cdot \mathbf {H}(\mathbf {F}) \end{aligned}$$

$\mathbf {C}(\mathbf {P})$ is a vector of coefficients that are functions of the power allocations ($\mathbf {P}$). Further, $\mathbf {H}(\mathbf {F})$ is a vector of basis functions that depend on the feature parameters ($\mathbf {F}$). We can determine $\mathbf {C}(\mathbf {P})$ by applying the method of least squares (or regression analysis), while using the pairs of measured $Perf(\mathbf {P}, \mathbf {F})$ and $\mathbf {H}(\mathbf {F})$—the details of this are explained in the next section. In addition, the definitions of $\mathbf {H}(\mathbf {F})$ used in our evaluation, which cover footprint awareness, are provided in Sect. 6.

## System Design

Based on the formulation/modeling provided in the last section, we introduce a system design to realize our approach. More specifically, we first explain the overview of our optimization framework and then describe our efficient calibration methodology to set the model coefficients. Finally, we provide our power allocation algorithm.

### Framework Overview

Figure 7 demonstrates our optimization methodology. Following the prior node-level power management studies [4, 34], we consider an application kernel-level power optimization. The library call start_power_opt() in the figure first collects the needed feature values ($\mathbf {F}$) and then distributes the allocated power budget to the components based on the obtained statistics. Here, we assume the library interacts with the system resource manager and receives the total power budget ($P_{sum}$), which is given as an environment variable and manually set in our evaluation. The library call end_power_opt() indicates the end point of the kernel, and thus the optimization finishes here. In addition, we acquire $\mathbf {F_{prof}}$ at this point during a profile run, which can be initiated by the user or is conducted when there is no profile for the application. On the other hand, scale/inputs dependent features ($\mathbf {F_{dy}}$), such as the footprint size ($F_{fs}$), need to be obtained at every execution.

**Fig. 9.**
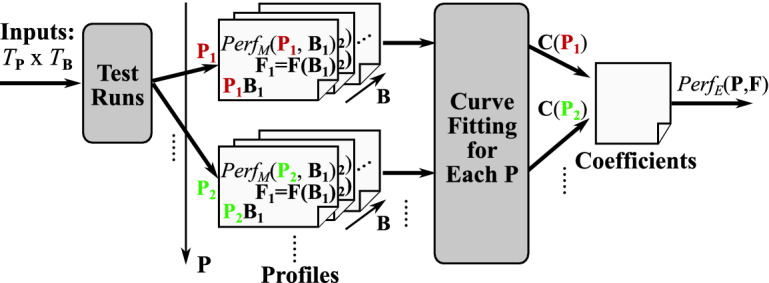
Model calibration overview

Figure 8 illustrates the workflow of our framework. Before using our power optimization approach, the offline calibration process is needed to determine the coefficients ($\mathbf {C}(\mathbf {P})$) in our model. This is conducted *only once for a system* by using a set of benchmarks, each of which consists of a kernel and inputs. Then, we optimize the power cap settings ($\mathbf {P}$) by using $\mathbf {C}(\mathbf {P})$ as well as $\mathbf {F}$ and $P_{sum}$ at runtime.

**Table 2.** Parameters/functions used in our calibration

Symbols	Remarks
$Perf_M(\mathbf {P}, \mathbf {B})$	Measured performance as a function of $\mathbf {P}$ and $\mathbf {B}$ (benchmark)
$Perf_E(\mathbf {P}, \mathbf {F})$	Estimated performance using our model: $Perf_E(\mathbf {P}, \mathbf {F})=\mathbf {C}(\mathbf {P})\cdot \mathbf {H}(\mathbf {F})$
$T_{\mathbf {P}} (\subseteq U_{\mathbf {P}})$	Set of tested power combinations: $T_{\mathbf {P}}=\{\mathbf {P_1}, \mathbf {P_2}, \cdots \}$, $\mathbf {P_j}= (P^j_{cpu}, P^j_{mem1}, \cdots$)
$T_{\mathbf {B}}$	Set of tested benchmarks: $T_{\mathbf {B}}=\{\mathbf {B_1}, \mathbf {B_2}, \cdots \}$, $\mathbf {B_k}= (Kernel_k, \mathbf {Inputs_k})$
$U_{\mathbf {P}}$	Set of all the power budget combinations: $U_{\mathbf {P}}=\{(P_{cpu}, P_{mem1}, \cdots ) | \forall P_{x}\in S_{P_{x}}\}$
$\mathbf {P^{max}}$	Maximum power cap settings: $\mathbf {P^{max}} = (P^{max}_{cpu}, P^{max}_{mem1}, \cdots )$, $P^{max}_{x}=\max (S_{P_{x}})$

### Efficient Coefficients Calibration

Figure 9 illustrates how we set the model coefficients appropriately through the calibration process. The inputs here are a set of power cap combinations ($T_{\mathbf {P}}$) and a set of benchmarks ($T_{\mathbf {B}}$). Then, we measure the performance ($Perf_M(\mathbf {P}, \mathbf {B})$) as well as the feature parameters ($\mathbf {F}$) for each power cap combination and each benchmark. By using these measured statistics, we identify the coefficients vector ($\mathbf {C}(\mathbf {P})$) for each power budget setting through the least-square curve fitting method. Then, we store the obtained coefficients in a file which is utilized at runtime to estimate the performance ($Perf_E(\mathbf {P}, \mathbf {F})$). Note that the definitions of functions/parameters used here are summarized in Table 2.

We determine all coefficients by only exploring a limited area of the entire space of all power cap combinations ($U_{\mathbf {P}}$) as examining all possible combinations for the calibration would be practically infeasible, especially for larger numbers of power caps and components. More specifically, we just scale the power cap value of one of the components turn-by-turn, obtain the coefficients for these power cap settings, and then estimate all coefficients for the entire power combination space by applying the following simple linear interpolation:

2 $$\begin{aligned} C_i(\mathbf {P})= & {} C_i(\mathbf {P^{max}}) + \left\{ C_i(P_{cpu},P^{max}_{mem1},P^{max}_{mem2},\cdots ) - C_i(\mathbf {P^{max}})\right\} \nonumber \\&+ \left\{ C_i(P^{max}_{cpu},P_{mem1},P^{max}_{mem2},\cdots ) - C_i(\mathbf {P^{max}})\right\} + \cdots \end{aligned}$$

**Fig. 10.**
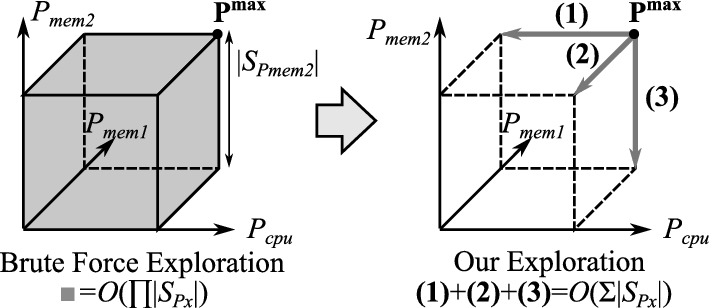
Efficient exploration in our calibration

Figure 10 illustrates how our approach improves the calibration efficiency in terms of the exploration space reduction. Although the brute force based naive exploration examines all the power cap combinations ($T_{\mathbf {P}}=U_{\mathbf {P}}$), ours just moves the space linearly. As a consequence, the number of tested power combinations is reduced significantly from $O(\prod |S_{P_x}|)$ to $O(\sum |S_{P_x}|)$.

**Fig. 11.**
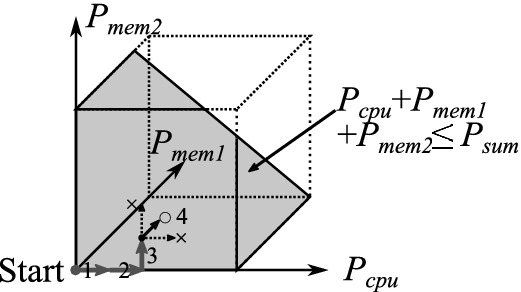
Hill climbing algorithm

### Power Allocation Algorithm

Next, based on the calibrated performance model, we optimize the power allocations for the running job under the given power constraint. As the brut-force approach searches for the best in the large number of combinations represented as $O(\prod |S_{P_x}|)$, which is practically infeasible, especially for larger numbers of power cap values and components, we alternatively consider an algorithm based on a hill climbing heuristic. The overview of the algorithm is illustrated in Fig. 11. We firstly set the power cap of each component at its minimum, and then we choose one and increase its power cap step-by-step while the total power cap meets the constraint. In each step, we select the component that improves the objective function the most with the one-step power cap increment. Although, the algorithm can finish at a locally optimal point, it does work well for monotonically increasing functions, such as performance, which increases with higher power cap allocations ($P_x$).

The precise form of our approach is described in Algorithm 1. The algorithm returns an estimated optimal power allocations vector ($\mathbf {P}$) for the given objective function, job features, and power constraint (*Obj*, $\mathbf {F}$, $P_{sum}$). The Lines 1 to 4 represent the initialization process: setting all power caps to minimums and sorting the set of power caps of each component in the ascending order. Then, the main loop follows after this—here, we increase the power caps of components step-by-step. In the inner-most loop (Line 7 to 13), we increase the power cap of each component by one step in each turn and register both its ID and the value of the objective function, if it meets all of the following conditions (Line 10): (1) the power cap did not reach the maximum in this previous; (2) the objective function returns the temporal optimum; and (3) the sum of the power caps is less than or equal to the power constraint. When this inner-most loop finishes, we decide whether we need to update the power cap combinations (Line 14 to 18). If the objective function value is improved in the above inner-most loop, we select the registered component and update its power cap by popping the front one from the associated power cap set; otherwise we just abort here. Finally, at the Line 20, we return the chosen power cap combinations.
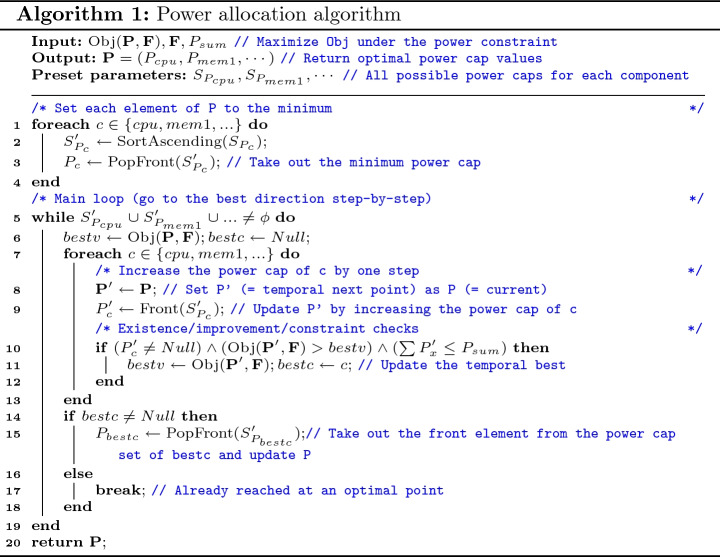

**Table 3.** System configurations

Name	Remarks
CPU Package	Xeon Gold 6154 Processor (Skylake) x2 sockets, 36 cores
Memory System	**DRAM (Memory 1):** DDR4-2666 x12 DIMMs, 12ch, 192 GB, 256 GB/s(max), **NVRAM (Memory 2):** Intel Optane SSD P4800X x2 cards, 750 GB, 4.8 GB/s (read max), 4.0 GB/s (write max), **Data management:** IMDT [14]
OS	Cent OS 7.4
Compiler	Intel C++/Fortran Compiler 17.0.4, **Options:** -O3 -qopenmp -xCORE-AVX512
Power caps[W]	$S_{p_{cpu}} = \{160, 170, \cdots , 280\}$, $S_{p_{mem1}}=\{20, 30, \cdots , 60\}$, $S_{p_{mem2}}=\{20, 30, 40\}$

## Evaluation Setup

**Environment: ** Our approach is applicable to any system that meets the following conditions: (1) the main memory is heterogeneous in terms of capacity and performance; and (2) component-wise power/performance controls are possible. In this evaluation, we use the platform summarized in Table 3, which follows the above conditions. As shown in the table, our main memory consists of DDR4 DRAM and PCIe attached NVRAM (Intel 3D Xpoint Optane [14]). By using Intel Memory Drive Technology (IMDT) [14], we can use the NVRAM as a part of the main memory^2^. More specifically, it works as a virtual machine monitor dedicated to the data management among the different kinds of memories, and these memories are used in a hierarchical manner: the DRAM is accessed first, and if it turns out to be a miss, then data swap happens (at page-level granularity). Note that our approach is applicable/extensible to any other emerging platforms with hybrid main memories such as 3D stacked DRAM + DIMM-based DRAM like Knights Landing [16] or DRAM + DIMM-based NVRAM like DCPMM [15], if they accept component-wise power managements. Only one thing we need to do to apply our method to them is just calibrating the model coefficients beforehand (or for finer tuning, adding/optimizing the basis functions for the target system is one option).

**Power Controls:** For the power management, we set various power cap values to the CPU and the DRAM through an interface based on RAPL (Running Average Power Limit) [13], which are listed in Table 3. Since power capping is not supported on our NVRAM, we emulate it by limiting the PCIe link speed (Gen1/2/3). More specifically, the link speed (Genx, x = 1, 2, 3) is selected so that the NVRAM power cap ($P_{mem2}$) fits the following:

3 $$\begin{aligned} P_{mem2}= & {} P_{dynamic}(x) + P_{static} + P_{margin}(x)\end{aligned}$$

4 $$\begin{aligned} P_{dynamic}(x)= & {} B_{link}(x)/B_{link}(3)*P_{dynamic}(3) \end{aligned}$$

The first equation ensures that the power cap value ($P_{mem2}$) is dividable into the dynamic power part ($P_{dynamic}$), the static power ($P_{static}$) and the accordingly set margin to round up ($P_{margin} < 10$[W]). The second equation ensures that the dynamic power limit is proportional to its link bandwidth ($B_{link}$). We use this because (1) the link speed limits the memory access frequency, and (2) the dynamic power consumption is, in principle, equal to the product of the energy consumption per access and the access frequency. We take $B_{link}(x)$, $P_{static}$ and $P_{dynamic}(3) + P_{static}$ from the official specs and determine the link speed for a given $P_{mem2}$. More specifically, we set the link as Gen1/2/3 for $P_{mem2} =$ 20/30/40 [W], respectively.

**Table 4.** Benchmarks ($T_{\mathbf {B}}$) used for our calibration

(Kernel, Inputs)
(miniFE, I1 = “-nx 512 -ny 512 -nz 512”), (miniFE, I2 = “ -nx 896 -ny 896 -nz 640”),
(miniFE, I3 = “-nx 1024 -ny 512 -nz 512”), (miniFE, I4 = “-nx 1024 -ny 768 -nz 640”),
(miniFE, I5 = “-nx 1024 -ny 1024 -nz 512”), (miniFE, I6 = “-nx 1024 -ny 1024 -nz 640”),
(LULESH, I1 = “-s 400”), (LULESH, I2 = “-s 450”), (LULESH, I3 = “-s 500”),
(LULESH, I4 = “-s 550”), (LULESH, I5 = “-s 600”), (LULESH, I6 = “-s 645”),
(MCB, I1 = “–nZonesX=2048 –nZonesY = 2048”), (MCB, I2 = “–nZonesX=4096 –nZonesY = 2048”),
(MCB, I3 = “–nZonesX=4096 –nZonesY = 3072”), (MCB, I4 = “–nZonesX=4096 –nZonesY = 4096”),
(MCB, I5 = “–nZonesX=5120 –nZonesY = 4096”), (MCB, I6 = “–nZonesX=6144 –nZonesY = 4096”),
(Streaming(AI: 10.7), I1 = “$N\,=\,16G$”), (Streaming(AI: 10.7), I2 = “$N\,=\,24G$”),
(Streaming(AI: 10.7), I3 = “$N\,=\,32G$”), (Streaming(AI: 10.7), I4 = “$N\,=\,48G$”),
(Streaming(AI: 10.7), I5 = “$N\,=\,64G$”), (Streaming(AI: 10.7), I6 = “$N\,=\,80G$”),
(AMG, I1 = “-n 512 512 256”), (AMG, I2 = “ -n 512 521 512”), (AMG, I3 = “-n 640 512 640”),
(AMG, I4 = “-n768 768 512”), (AMG, I5 = “-n 640 640 640”), (AMG, I6 = “-n 1024 640 512”),
(Streaming(AI: 0.167), I1 = “$N\,=\,16G$”), (Streaming(AI: 0.167), I2 = “$N\,=\,24G$”),
(Streaming(AI: 0.167), I3 = “$N\,=\,32G$”), (Streaming(AI: 0.167), I4 = “$N\,=\,48G$”),
(Streaming(AI: 0.167), I5 = “$N\,=\,64G$”), (Streaming(AI: 0.167), I6 = “$N\,=\,80G$”)

**Table 5.** Problem settings for our power allocation evaluation

Application	[Problem]: (Inputs, Footprint Size[GB])
miniFE	[Small]: (“-nx 1024 -ny 512 -nz 512”, 129), [Large]: (“-nx 1024 -ny 1024 -nz 640”, 321)
LULESH	[Small]: (“-s 400”, 62), [Large]: (“-s 645”, 258)
MCB	[Small]: (“–nZonesX = 2048 –nZonesY = 2048”, 57), [Large]: (“–nZonesX = 5120 –nZonesY = 4096”, 279)
AMG	[Small]: (“-n 512 512 512”, 141), [Large]: (“-n 1024 640 512”, 354)
Stream(AI:*)	[Small]: (“$N\,=\,8G$”, 64), [Large]: (“$N\,=\,32G$”, 256)

**Methodology: ** To evaluate our approach, we use the synthetic code (Streaming) shown in Fig. 2 (Sect. 3.1) as well as several mini applications chosen from the CORAL benchmark suite [25]: AMG, LULESH, MCB and miniFE. For each application, we regard the main loop as a target kernel. The benchmark set ($T_{\mathbf {B}}$) used for our calibration process is listed in Table 4; we test various inputs for each application kernel. Then, by using the obtained coefficients, we optimize the power allocations for the workloads listed in Table 5. Here, the data footprint fits within the fast memory (192[GB]) for Small problems, but it does not for Large problems.

Next, Table 6 describes the feature parameters ($\mathbf {F}$) utilized in our evaluation. On one hand, we measure $\mathbf {F_{dy}}$ at every run, while on the other hand, we collect $\mathbf {F_{prof}}$ only once for an application, especially with the Small problems shown in Table 5. By using PAPI [37], we collected these feature parameters^3^. Note that, through our preliminary evaluation, we confirmed that all of $\mathbf {F_{prof}}$, including the LLC (Last Level Cache) access statistics ($F_{p3}$ and $F_{p4}$), are almost constant when we scale the problem sizes from few GiB to few 100 GiB for these applications, thus we consider them as scale-independent, yet application-specific parameters in this work.

**Table 6.** Feature parameter selections ($\mathbf {F}=(\mathbf {F_{prof}}, \mathbf {F_{dy}})$)

Types	Parameter remarks
$\mathbf {F_{prof}}$	$F_{p1}$=(# of FP operations)/(# of instructions), $F_{p2}$ = (# of non FP arithmetic instructions)/(# of instructions), $F_{p3}$ = (# of LLC misses)/(# of instructions), $F_{p4}$ = (# of LLC misses)/(# of LLC accesses),
$\mathbf {F_{dy}}$	$F_{d1}$ = (footprint Feature parameter selections size $F_{fs}$)/(capacity of Memory1)

**Table 7.**
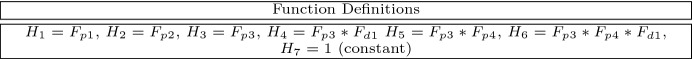
Basis function setups ($\mathbf {H(F)}=(H_1(\mathbf {F}), H_2(\mathbf {F}), \cdots )$)

Table 7 shows the list of the basis functions ($\mathbf {H}(\mathbf {F})$) utilized in our evaluation. By using $H_1$ and $H_2$, we detect the CPU load and how much it affects the power capping settings. In addition to them, we also consider the traffic on the overall hybrid memory system and how each of them are accessed by using the functions $H_{3}$, $H_{4}$, $H_{5}$, and $H_{6}$. Because $F_{p3}$ is equal to the frequency of accesses to the memory system, $H_{3}$ indicates how heavily it is used. In addition, we utilize $F_{p4}$ and/or $F_{d1}$ for $H_{4}$, $H_{5}$ and $H_{6}$ due to the following reasons: (1) because the LLC hit rate $F_{p4}$ is sensitive to the memory access pattern, we can use it to cover this aspect; (2) to take problem scale into account, we further utilize $F_{d1}$ here as well. These parameters are multiplied by $F_{p3}$ as the impacts of access-pattern/problem-scale on performance depend on the access frequency, and we thus take the correlation of these parameters into consideration.

Although this selection of parameters and the function settings are effective, as shown in the next section, it may be possible to further improve the accuracy by consider additional aspects. For instance, adding other memory-access related parameters, such as working-set size, could be a good option for workloads with more complicated inputs. We can provide such an extensibility in a straightforward manner by making the model parameters/terms modifiable by users and then making them available to the other parts of the framework, like calibration and power allocation.

**Fig. 12.**
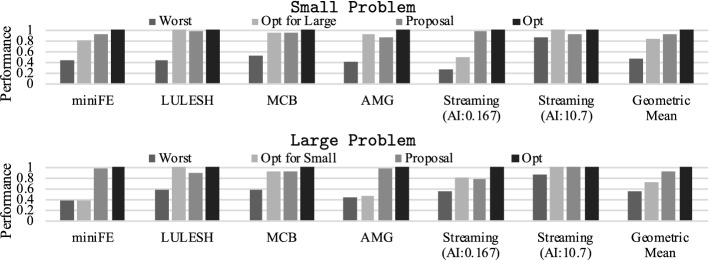
Performance comparisons at $P_{sum}=300$[W] for different problem sizes (U: Small, D: Large)—the objective function for our approach is *Perf*()

**Fig. 13.**
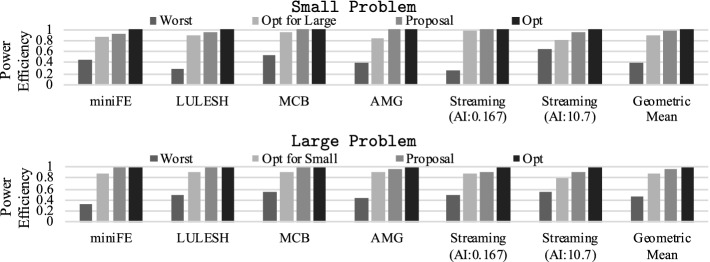
Power-efficiency comparisons at $P_{sum}=300$[W] for different problem sizes (U: Small, D: Large)—the objective function for our approach is *PowEff*()

**Fig. 14.**
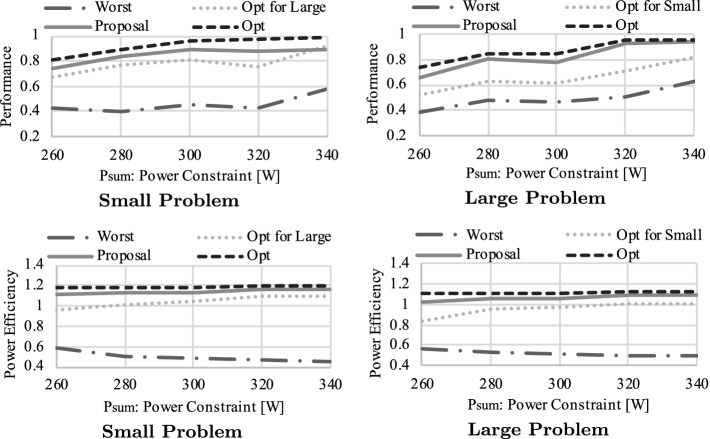
Performance (U) and Power efficiency (D) as functions of the node power constraint for different problem sizes

## Experimental Results

In Figs. 12 and 13, we compare performance/power-efficiency across methods using different problem sizes. Here, we set $P_{sum}$ to 300[W] and utilize *Perf*()/*PowEff*() as the objective function in our approach through the measurements of Figs. 12 and 13. The vertical axis indicates relative performance or power-efficiency, normalized to the optimal power cap combinations that maximize the given objective function. The *Worst* combination is chosen from the settings that meet $\sum P_{x}=P_{sum}$ or $\sum P_{x}\le P_{sum}$ in Fig. 12 or Fig. 13 so that the objective function is minimized^4^. GeometricMean indicates the geometric mean of performance or power efficiency across all workloads for each method. Overall, our approach achieves near optimal performance/power-efficiency: on average, our approach keeps 93.7%/96.2% or 92.3%/95.4% of performance/power-efficiency compared to the optimal for Small or Large problems. Note that these numbers are quite important as we consider the situation where the power scheduler distributes power budgets to the nodes, and each node needs to optimize the power allocations to the components while keeping the given power constraint, which is regarded as common in future power-constrained supercomputers.

Then, we scale the total power budget ($P_{sum}$) and observe performance and power efficiency for all the above methods. In Fig. 14, we summarize the experimental result using the geometric mean of performance/power-efficiency across all workloads. In the graphs, the X-axis indicates the node power constraint ($P_{sum}$), while the Y-axis shows relative performance or power efficiency normalized to the maximum power cap setting ($\mathbf {P}=\mathbf {P^{max}}$). As shown in the figures, our approach is very close to the optimal regardless of the problem size, the objective function, or the total power budget.

**Fig. 15.**
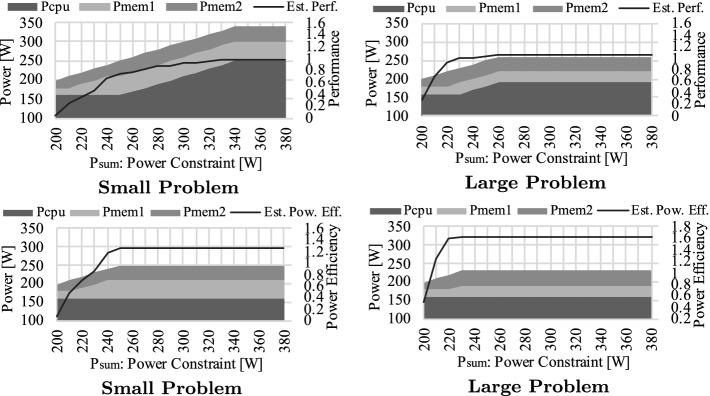
Power cap settings determined by our approach for miniFE

**Fig. 16.**
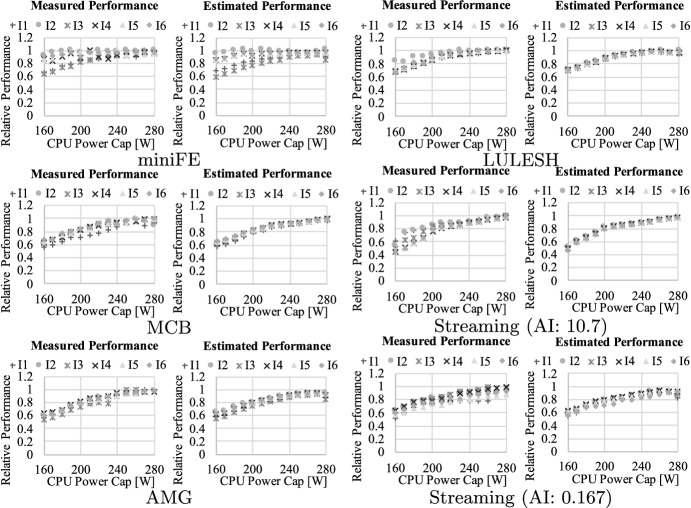
Comparison of measured (left) and estimated (right) performance for different $P_{cpu}$ ($P_{mem1}=P^{max}_{mem1}=60[W]$, $P_{mem2}=P^{max}_{mem2}=40[W]$)

Next, we demonstrate how our approach distributes the given power budget ($P_{sum}$) depending on several aspects by using miniFE as an example. Figure 15 illustrates the breakdowns of power allocations in accordance to the given power constraint ($P_{sum}$) as well as the objective function for different problem sizes (Small/Large). The horizontal axis represents the power constraint ($P_{sum}$), while the vertical axis indicates the breakdown or relative performance/power-efficiency normalized to $\mathbf {P}=\mathbf {P^{max}}$. Note that the performance or power-efficiency curves in the figures are the estimated values provided by our model, and the allocations are based on them.

According to the figures, even for the same application, the power allocation decisions can change considerably depending on the objective function as well as the problem settings. For Small, our method initially allocates power to the memory system side and then shifts to the CPU side until reaching 340[W] to maximize performance (upper left figure). However, when the problem size is scaled, the CPU and the first memory need less power. This is because the second memory becomes the significant bottleneck, and allocating more power to the others does not help with improving performance (upper right figure). As for the power efficiency (lower figures), our approach stops the power allocations earlier because it requires large enough performance gain that is worthwhile putting additional power. For most of the evaluated workloads, we also observe the exact same situation: the given power budget cannot be fully used, especially when the problem size is scaled. We regard this as an opportunity to improve the whole system efficiency (e.g., by returning such extra power budget to the system manager and allocating it to other jobs).

**Fig. 17.**
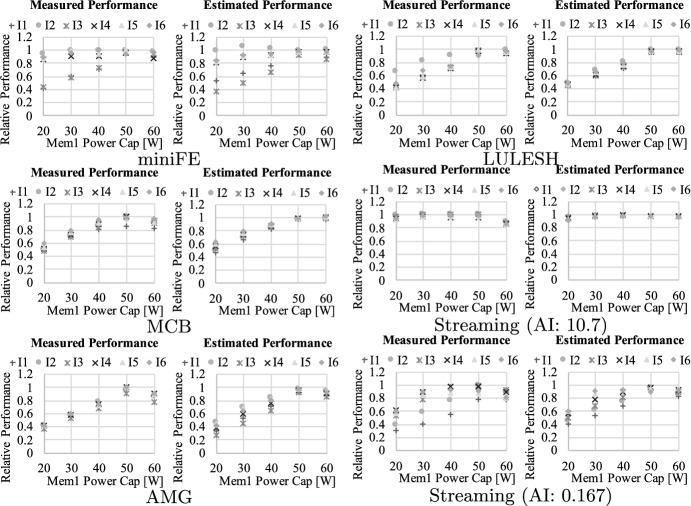
Comparison of measured (left) and estimated (right) performance for different $P_{mem1}$ ($P_{cpu}=P^{max}_{cpu}=280[W]$, $P_{mem2}=P^{max}_{mem2}=40[W]$)

**Fig. 18.**
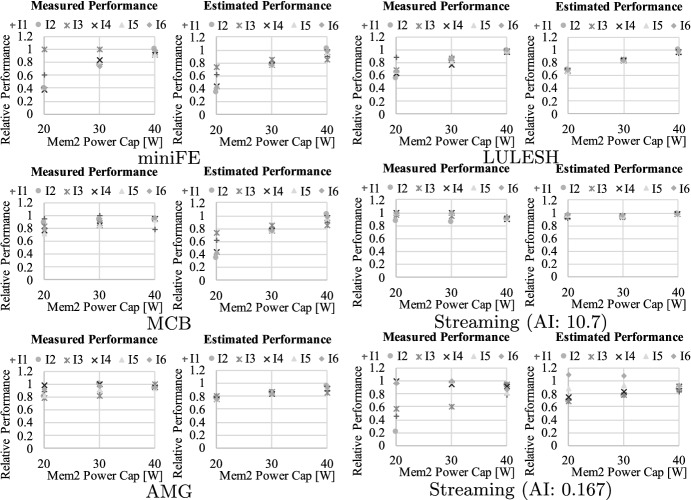
Comparison of measured (left) and estimated (right) performance for different $P_{mem2}$ ($P_{cpu}=P^{max}_{cpu}=280[W]$, $P_{mem1}=P^{max}_{mem1}=60[W]$)

Further, in Fig. 16, 17, and 18, we demonstrate the model calibration result using the workloads described in Table 4. For each graph, the horizontal axis indicates the power capping value set at each component, while the vertical axis represents relative performance which is normalized to that at best—namely, setting $\mathbf {P}$ at $\mathbf {P^{max}}$. Each legend is associated with the problem (or inputs) settings shown in Table 4. Here, we applied the method of least squares using sets of relative performance and feature parameters brought by the workloads. Overall, our approach successfully captures the characteristics of these applications including the footprint size dependency, and the estimated result is close to the measured performance for almost all the cases (the average error is only 6.00%).

Finally, we measured the time overhead of our approach, which turned out to be negligible. More specifically, it took only around 200 ${\upmu }$s, 1 ${\upmu }$s, and 80 ${\upmu }$s for accessing feature parameters through PAPI, conducting our decision algorithm (completed at $\mathbf {P}=\mathbf {P^{max}}$), and setting a power cap through RAPL, respectively.

## Conclusions

In this article, we firstly focused on the bottleneck shifting phenomenon when scaling the problem size on a real system that consists of a hybrid main memory. Based on this observation, we introduced the concept of  and demonstrated its potential benefit using various HPC benchmark applications. Motivated by this preliminary result, we defined the problem, formulated a solution and provided a software framework to realize our concept. Finally, we quantified the effectiveness of our approach, which showed that it achieves near optimal performance/power-efficiency.

As a next-step, we will evaluate our approach using more complicated real-world applications and show the effectiveness with them. Another direction will be the coordination between our framework and a power scheduler to optimize both intra- and inter-node power budget settings at the same time. We expect that this will have a significant impact on full system energy efficiency, as the power budget to a node is prone to be under-utilized when the footprint size is large. Consequently, sending this as feedback to the power scheduler will help whole system performance/energy-efficiency under the total power constraint. Another promising direction is an extension of our work to cover other kinds of systems (e.g., CPU + GPU/FPGA + hybrid memory) or other application areas, such as data analytics or machine learning using various types of hybrid memories. Although we may have to update the parameters/terms of the regression model, the concept of FPCAP and the approaches used in our framework will carry forward and improve system efficiency.

## Footnotes

1 This phenomenon could happen on traditional systems using monolithic main memories when the footprint size were in the neighborhood of ***the on-chip cache capacity*** (at most few 10 MB), which is not the case for HPC applications in general.

2 Persistent Memory Development Kit (PMDK) also supports an automatic data management feature and can be used for this purpose [1].

3 We disable IMDT when collecting $\mathbf {F_{prof}}$ as it prevents us from accessing hardware counters. But, this is not the case when we use PMDK for the data management.

4 If we choose the worst of $\{\forall \mathbf {P} | \sum P_{x}\le P_{sum}\}$ for the performance evaluation, it will always mean setting all power caps to the minimum. Therefore, we set the constraint as $\sum P_{x}=P_{sum}$ for Worst in the performance evaluation.
